# Invasive Meningococcal Disease in a Patient With Complement 7 Deficiency

**DOI:** 10.1002/jgf2.70075

**Published:** 2025-10-15

**Authors:** Hiroaki Nishioka, Kazuki Maegawa, Shigeo Hara

**Affiliations:** ^1^ Department of General Internal Medicine Kobe City Medical Center General Hospital Kobe Japan; ^2^ Department of Pathology Kobe City Medical Center General Hospital Kobe Japan

**Keywords:** complement 7 deficiency, invasive meningococcal disease, leukocytoclastic vasculitis, *Neisseria meningitidis*, purpura

## Abstract

*Neisseria meningitidis* can cause invasive meningococcal disease (IMD). Individuals with primary complement deficiencies are at a higher risk of developing IMD. However, cases of IMD associated with complement deficiency have rarely been reported in Japan. In this case, a 35‐year‐old Japanese man presented with fever and a spreading skin rash. Blood culture identified *N. meningitidis* serogroup B. The patient was treated with antibiotics and fully recovered. Genetic analysis revealed a deficiency in complement component 7. This case highlights that IMD can occur in young, otherwise healthy individuals, some of whom may have underlying complement deficiency.

## 1 Background

*Neisseria meningitidis* is a gram‐negative diplococcus colonizing the nasopharynx of healthy individuals, particularly adolescents and young adults. *N. meningitidis* spreads via respiratory droplets and can cause invasive meningococcal disease (IMD), presenting as meningitis and/or septicemia. *N. meningitidis* has 12 serogroups, with six (A, B, C, X, Y, W) linked to IMD. IMD progresses rapidly, often within 24–48 h. Severe cases can progress to purpura, hypotension, and multi‐organ failure, with mortality of 10%–15% [1]. IMD often affects young, healthy individuals, with the complement system as the first defense. Susceptibility to IMD is associated with defects in the alternative complement pathway components (such as properdin and factor D) and terminal complement pathway (C5–C9) [2]. The increasing use of C5 inhibitors (eculizumab, ravulizumab) has raised concerns regarding IMD vulnerability. Few cases of primary complement deficiency have been reported in Japan [3, 4].

Leukocytoclastic vasculitis is a small‐vessel vasculitis characterized by leukocytoclasis and can occur in various conditions, including antineutrophil cytoplasmic antibody‐associated vasculitis, connective tissue disease, and infections. Only a few cases of leukocytoclastic vasculitis have been reported in patients with meningococcal disease, mostly chronic infections [5]. Acute meningococcal disease with leukocytoclastic vasculitis is rare.

In this report, we present the case of a Japanese man who developed IMD accompanied by leukocytoclastic vasculitis without immune complex deposition and was subsequently diagnosed with complement 7 (C7) deficiency based on genetic analysis.

## 2 Case Presentation

**FIGURE 1:**
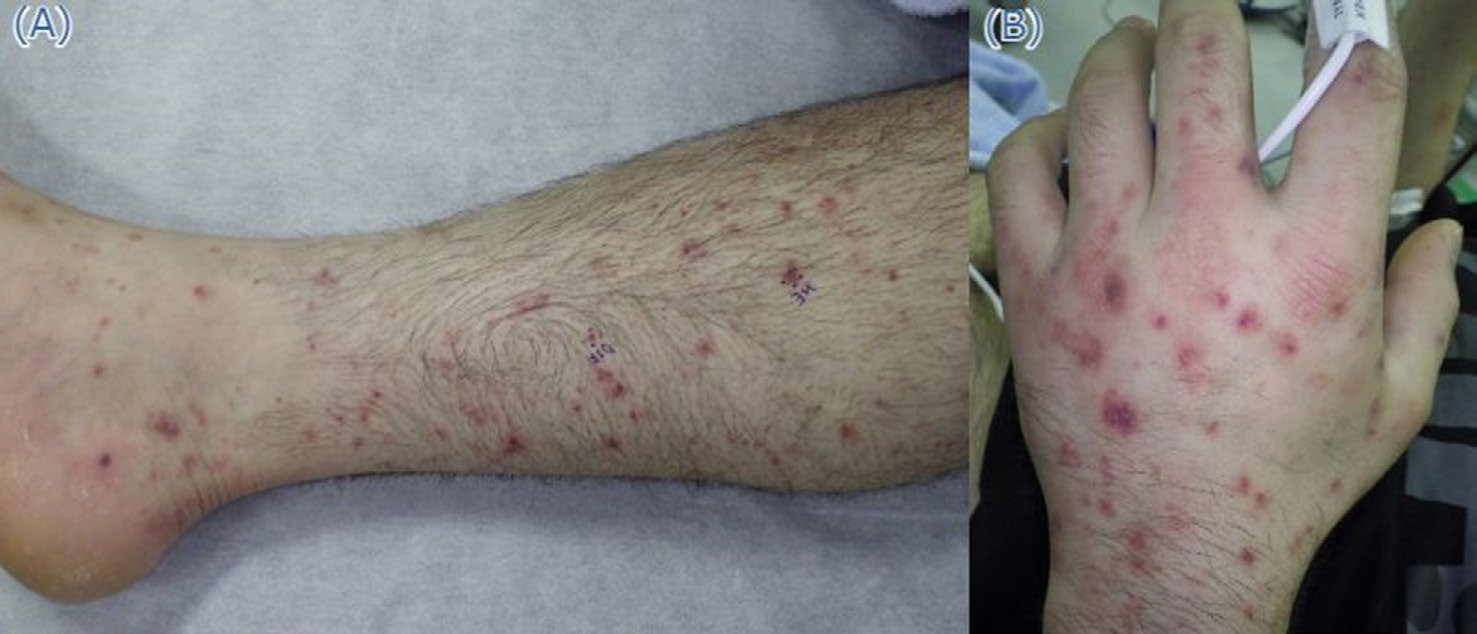
Physical examination findings. Purpura is observed in the lower leg (A) and the back of the hand (B).

**FIGURE 2:**
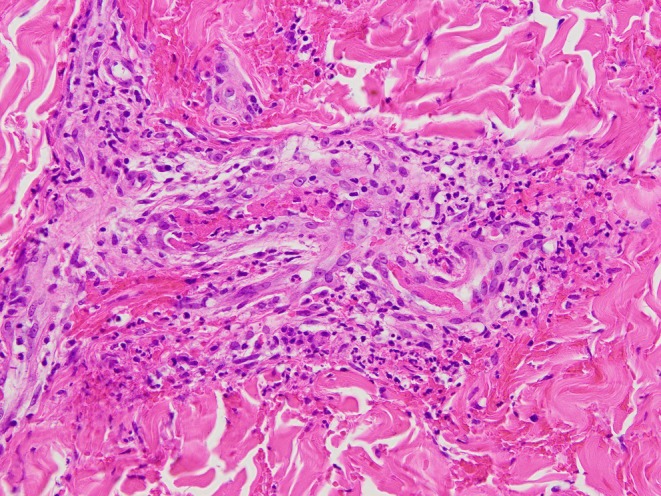
Histopathological findings of the purpura. Infiltration of inflammatory cells, primarily neutrophils and lymphocytes, and hemorrhage were observed around small blood vessels. This was accompanied by nuclear fragmentation and fibrin deposition, indicative of leukocytoclastic vasculitis.

A 35‐year‐old Japanese man presented with a two‐day history of fever, polyarthralgia, and an expanding skin rash. He had no medical history, was not on any medications, had not traveled overseas, and had no contact with sick individuals. Examination showed a temperature of 37.7°C, blood pressure of 122/74 mmHg, and heart rate of 105 bpm. The patient was alert and conscious, with a Glasgow Coma Scale of E4V5M6, and reported no headache. No nuchal rigidity was observed. The conjunctivae were hyperemic with eye discharge. Purpura was noted in the extremities and trunk (Figure 1A,B). The bilateral wrist, metacarpophalangeal, and ankle joints were swollen and tender, with a limited range of motion. The chest and heart sounds were normal. Laboratory findings were as follows: white blood cell count, 9100/μL; platelet count, 73,000/μL; aspartate aminotransferase, 99 U/L; alanine aminotransferase, 169 U/L; C‐reactive protein, 21.2 mg/dL; erythrocyte sedimentation rate, 89 mm/h. Tests for rheumatoid factor, antinuclear antibodies, myeloperoxidase‐antineutrophil cytoplasmic antibody (ANCA), and proteinase 3‐ANCA returned negative results. While C3 and C4 levels were normal, the 50% hemolytic complement activity (CH50) level was low, measuring less than 10.0/mL. Contrast‐enhanced computed tomography showed no abnormalities, and joint ultrasonography revealed synovial proliferation and inflammation. Ceftriaxone (1 g every 12 h) was administered due to a suspected infection. On day 2, two sets of blood cultures obtained on admission tested positive for *N. meningitidis* 18 h after the blood was collected. Serogroup typing and multilocus sequence typing classified the isolate as serogroup B and genetic type ST‐2057, respectively. Skin biopsy of leg purpura showed leukocytoclastic vasculitis without C1q, C3, C4, IgA, IgG, or IgM deposition, as confirmed by direct immunofluorescence (Figure 2). By day 3, the patient's fever subsided. The rash faded, and the joint pain improved. On day 6, the antibiotic was changed to ampicillin (2 g every 4 h) based on the results of an antimicrobial susceptibility test. He received antibiotics for 10 days and was subsequently discharged. Reexamination of the CH50 level showed that it was low again (< 10/mL).

Given that the patient developed a meningococcal infection and had a low CH50 despite normal C3 and C4 levels, we considered the possibility of a primary complement deficiency. We analyzed the genomic sequence of the protein‐coding exon of the complement gene and its boundary region by examining 10 bases inside the adjacent intron using a target‐capture method for next‐generation sequencing. A large heterozygous deletion of the entire C7 gene was detected, leading to a diagnosis of complement C7 deficiency. Future vaccinations were recommended.

## 3 Discussion

The clinical course of our patient highlights two important observations. First, IMD can affect previously healthy young individuals, some of whom may have complement deficiencies. Second, leukocytoclastic vasculitis may accompany acute meningococcal infection.

The incidence of IMD varies globally, ranging from 1 to 1000 cases per 100,000 individuals. In sub‐Saharan Africa, explosive epidemics have resulted in over 30,000 reported cases of IMD annually. In Japan, 30–40 IMD cases occur annually, with serogroup B being second to Y. This difference may reflect Japan's lower *N. meningitidis* carriage rate (0.4%–0.8%), which is far below that of many other countries [6]. Universal health insurance in Japan enables prompt access to medical care. Patients with terminal complement defects have a 7000–10,000‐fold higher IMD risk, and more than half experience recurrence [2]. However, few IMD cases associated with primary immunodeficiency have been reported in Japan [3, 4]. The rarity of these evaluations may be attributed to the limited inclusion of routine complementary assessments in national surveys. In patients with complement deficiency, IMD is often associated with a milder form of the disease. This may be due to a less intense inflammatory response that occurs when the complement pathway is not fully functional. However, patients with complement deficiency are at high risk for infections from IMD and have a high recurrence rate. Therefore, complement testing is essential to guide vaccination and prophylaxis. The Centers for Disease Control and Prevention recommends two types of meningococcal vaccines: the MenACWY vaccine, which targets serogroups A, C, W, and Y, and the MenB vaccine, which targets serogroup B [7]. While the MenACWY vaccine was approved in Japan in 2015, there is currently no approved MenB vaccine available. Both vaccines elicit strong immune responses in both children and adults. However, patients with complement deficiency may still develop IMD even after achieving high antibody levels following vaccination, due to a lack of a functional complement pathway [8]. For patients receiving C5 inhibitors, it is recommended to administer daily antimicrobial prophylaxis with oral penicillin. Additionally, it may also be important to consider prophylactic oral antibiotics for patients with other types of complement deficiencies.

C7 deficiency is the second most frequent complement deficiency in Japan, after C9 deficiency, affecting approximately one in 10,000 individuals [9]. The key functions of the complement system are opsonization by C3. The membrane attack complex (MAC), formed from the C5–9 complex, contributes to immune protection by disrupting the pathogen membrane. Other bacteria can be cleared via C1–4 opsonization and neutrophil killing; however, *Neisseria* resist and survive intracellularly. Moreover, deficiencies in terminal complement components (C5–8) inhibit MAC formation and increase susceptibility to *Neisseria* infections.

Purpura is a common manifestation of meningococcal infection. Some cases have documented histological findings, including IgA vasculitis and septic vasculopathy [10, 11]. Few meningococcal cases with leukocytoclastic vasculitis lacking immune complex deposition have been reported, mostly in chronic infections [5]. Leukocytoclastic vasculitis associated with acute meningococcal diseases is considered rare. Leukocytoclastic vasculitis is characterized by neutrophilic infiltration, fibrinoid necrosis, and nuclear debris. It is linked to viral infections (hepatitis B/C, cytomegalovirus, and parvovirus B19) and bacterial infections (*Staphylococcus aureus, Streptococcus* spp., *Klebsiella pneumoniae* , and *Mycobacterium tuberculosis* ). Pathogen invasion may trigger excess nitric oxide and COX‐2, generating prostaglandins and oxidative stress, which damage vessels. In adult patients with meningococcal purpura fulminans, skin biopsy with conventional culture and meningococcal polymerase chain reaction demonstrated a global sensitivity of 88%, indicating the presence of the causative pathogen in acute skin lesions [12]. While rash is a common symptom of meningococcal disease, there is limited histopathological data available. A larger case series is needed to clarify the pattern and pathogenesis of the findings. This is expected to include an investigation into genetic factors, the virulence specific to serogroup B, the characteristics of the ST‐2057 strain, and other mechanisms that may affect hosts with complement deficiencies.

## 4 Conclusion

This case highlights that IMD can affect young, healthy individuals, some of whom may have complement deficiency. This underscores the importance of evaluating complement status in young adults who develop IMD, as these deficiencies can increase the risk of recurrence and necessitate preventive measures. Additionally, when treating patients with leukocytoclastic vasculitis that lacks immune complex deposition, it is important to consider acute meningococcal disease as a potential diagnosis.

## Author Contributions

All authors conceptualized the study. H.N. and K.M. prepared the original draft. H.N. and S.H. conducted visualization. H.N. managed and supervised the project. All authors have reviewed and edited the draft, and approved the final version of the manuscript.

## Disclosure

We have not utilized Artificial Intelligence Generated Content tools, such as ChatGPT and others, based on large language models.

## Ethics Statement

According to our institutional policy, ethics committee approval was not required for this case report.

## Consent

Written informed consent was obtained from the patient for publication of this case report and accompanying images.

## Conflicts of Interest

The authors declare no conflicts of interest.

## Data Availability

Data sharing is not applicable to this article because no datasets were generated or analyzed in this study.
